# Evidence That Pervasive Body Gaze Behavior in Heterosexual Men Is a Social Marker for Implicit, Physiological, and Explicit Sexual Assault Propensities

**DOI:** 10.1007/s10508-024-02953-y

**Published:** 2024-07-24

**Authors:** Ross C. Hollett, Hannah West, Candice Craig, Lorna Marns, James McCue

**Affiliations:** Psychology and Social Sciences, Edith Cowan University, 270 Joondalup Drive, Joondalup, WA 6027 Australia

**Keywords:** Body gaze, Objectification, Sexual assault, Rape myth, Physiological, Implicit Association Test

## Abstract

Deliberate and effortful attempts to gaze at the bodies of women is emerging as a valuable marker of sexual objectification in men. Some preliminary evidence suggests that pervasive body gaze behavior may also accompany insidious attitudes which can facilitate sexual assault. The present study aimed to further explore this potential by examining pervasive body gaze associations with explicit, implicit, and physiological sexual assault propensity measures. We presented 110 heterosexual male participants with images of fully and partially dressed women with and without injuries while measuring their skin conductance responses. We also captured implicit and explicit sexual assault measures in addition to self-reported pervasive body gaze behavior. Pervasive body gaze behavior was significantly correlated with rape myth acceptance attitudes, prior perpetration of sexual assault, a stronger implicit association between erotica and aggression, and lower physiological reactivity during exposure to partially dressed injured women. These findings suggest that body gaze towards women could be a behavioral marker for inclinations to victim blame, preferences for rough sexual conduct, and a physiological desensitization towards female victims. This study further validates a five item self-reported body gaze measure as a valuable tool for detecting deviant sexual objectification attitudes and affective states. As such, measurement and observation of body gaze behavior could be useful for developing risk assessments, estimating intervention efficacy, and enhancing public awareness.

**Supplementary Information:**

The online version contains supplementary material available at 10.1007/s10508-024-02953-y.

## Introduction

Sexual assault of women by men is a prolific and harmful social problem in developed countries such as Australia and the US, with estimates suggesting that between 15 and 20% of women have been victims of sexual assault in their lifetime (Australian Institute of Health & Welfare, 2020; Smith et al., 2018). Measuring the sociocognitive attitudes, inclinations, and behaviors which precede or accompany sexual assault is valuable for estimating and reducing the propensity to offend (Davis et al., 2014; Gervais et al., 2018; Riemer et al., 2022). However, such attitudes, inclinations, and behaviors are often difficult to measure with high confidence. One potentially promising and readily observable social marker for sexual assault propensity is pervasive body gaze behavior, characterized as effortful and uninhibited attempts to gaze at other people’s bodies (Hollett et al., 2022). Recent research has shown that self-reported pervasive body gaze behavior is positively correlated with objectively measured body gaze behavior and victim blaming attitudes in heterosexual men (Hollett et al., 2022). These preliminary findings suggest that pervasive body gaze, when measured via self-report, could be utilized to assess sexual assault propensity without specifically referring to sexual assault. For a psychometric tool (self-report or otherwise) to provide substantive value in understanding human intentions and behavior, considerable evidence of construct validity is required. Consequently, the purpose of the present study was to provide further evidence that self-reported pervasive body gaze is a valid maker of sexual objectification by investigating its correspondence with cognitive and affective markers of sexual aggression.

### Sexual Objectification of Women

Sexual objectification involves a process whereby a person’s intellectual, social and emotional attributes are devalued relative to their sexual body parts and functions, consistent with what occurs during a sexual assault (Fredrickson & Roberts, 1997; Hollett et al., 2022; Pecini et al., 2023; Riemer et al., 2022). Research has offered several consequences of sexual objectification which can partly explain why women are often mistreated as sexual objects. Specifically, when women’s bodily and sexual attributes are prioritized, they are at risk of being dehumanised, perceived as incompetent, denied moral concern, and viewed as culpable when they are sexually assaulted (Bernard et al., 2015; Heflick & Goldenberg, 2009; Loughnan et al., 2014; Loughnan et al., 2013; Pecini et al., 2023). It is widely agreed that these consequences of sexual objectification are partly perpetuated by entertainment, retail and social media which present women as appreciated for their appearance and sexual functions instead of their intellectual, social, and emotional characteristics (Aubrey & Frisby, 2011; Davis, 2018; Galdi et al., 2013; Hollett & Challis, 2023; Hollett et al., 2023; Lynch et al., 2016; Mikorski & Szymanski, 2017; Stankiewicz & Rosselli, 2008). That is, if women are repeatedly evaluated via their physical properties, then they are at greater risk of being used for sexual gratification with diminished remorse (Riemer et al., 2022).

In developed societies where technology facilitates high accessibility to sexually objectifying visual media, it could be argued that the sexual objectification of women has become a harmful social norm (e.g., Gervais & Eagan, 2017). This assumption is supported by high incidences of sexual harassment against women in these societies (Cassino & Besen-Cassino, 2019; Kahsay et al., 2020; Mennicke et al., 2022; Mumford et al., 2020). Visual media which highlights women’s bodies and sexual functions is also known to elicit sexually objectifying gaze behavior among men, or a preference to gaze at bodies or body parts (Gervais et al., 2013; Hollett et al., 2019, 2022; Karsay et al., 2018). Habituated attentional biases towards women’s bodies following repeated exposure to sexually objectifying media can partly explain why body biased gaze may subsequently occur towards real people. Body biased gaze (sometimes termed “male gaze”) is often identified as a behavioral manifestation of sexual objectification because it communicates an overemphasis on bodily features instead of intellectual, social, and emotional features when evaluating women (Fredrickson & Roberts, 1997; Gervais et al., 2013, 2018, 2020). That is, a person who increasingly allocates attentional resources towards evaluating women’s bodies is at risk of perceiving women as sexual objects. If body biased gaze behavior is a valid marker of sexual objectification, then it may also serve as a predictor of more sinister manifestations of sexual objectification, such as sexual assault. For instance, a person who commits a sexual assault against a woman is also likely to exhibit body biased gaze behavior when evaluating their potential victims. The measurement of body gaze, among other socio-cognitive and behavioral predictors of sexual assault, have attracted substantial interest, particularly in the development of self-report instruments.

### Self-Reported Measurement of Sexual Objectification

A large body of literature has attempted to capture the social norms and attitudes which facilitate harmful consequences of sexual objectification, such as sexual assault. One of the most popular conceptual frameworks for measuring sexual assault attitudes was posited by Burt (1980), which involved a set of cultural beliefs specific to the context of male perpetrated sexual assault against women, known as rape myths. Psychometric evidence suggests that the rape myth construct is broad. For example, the Illinois Rape Myth Acceptance Scale (IRMAS) developed by Payne et al. (1999) contained seven subscales related to victim blame (two subscales), perpetrator exoneration, rape triviality, rape deviance, female distrust, and misidentification of rape. Victim and perpetrator blame have been popular subconstructs within this framework for investigating experimental effects of exposure to sexually objectifying material (Beck & Rose, 2021; Bernard et al., 2015, 2018; Loughnan et al., 2013; Noël et al., 2021). In correlational research, the most thoroughly investigated sexually objectifying material has been pornography, arguably the epitome of sexually objectifying visual media (Fritz & Paul, 2017; Hedrick, 2021; Klaassen & Peter, 2015). Self-reported rape myth acceptance attitudes have been heavily utilized when attempting to quantify the socio-cultural effects of pornography. However, meta-analytic evidence suggests that the association between pornography and sexual assault attitudes is generally modest (e.g., *r* < .20) (e.g., Allen et al., 1995; Burnay et al., 2022; Hald et al., 2010). Regardless of the tentative links between sexually objectifying media use and rape attitudes, rape myth acceptance attitudes have been shown to predict intentions to rape (Taschler & West, 2017) and perpetration of male-to-female sexual violence (Tharp et al., 2012; Yapp & Quayle, 2018). Thus, the measurement of rape myth acceptance attitudes continues to be useful when attempting to validate other sexual objectification measures.

While the rape myth framework has been useful for exploring some aspects of sexually objectifying attitudes, recent work has focused on developing scales more consistent with theoretical descriptions of the sexual objectification construct (Gervais et al., 2018; Riemer et al., 2022). Importantly, these scales recognise that body gaze is an important marker for sexual objectification, often measured alongside items which also capture sexual assault behaviors. That is, the factor structure of these instruments suggest that body gaze behavior is part of the same general sexual objectification construct as sexual assault. One drawback of these scales is that some items refer directly to sexually aggressive or intimate contexts (e.g., grabbing/pinching private areas, hurting women during sex), which can be confronting, imply admission to criminal offenses, and are thus more ethically challenging to deploy. Admitting to body biased gaze behavior may offer similar psychometric and predictive benefits to more confronting scales with lower emotive cost. However, sexually objectifying gaze has been measured in differing ways. For instance, Gervais et al. (2018) used several highly repetitive body gaze items (e.g., stared, leered, gazed) to estimate the frequency by which body gaze occurs towards generic subjects (e.g., men or women). By contrast, Riemer et al. (2022) included only one body gaze item within a broader “appearance-based” objectification subscale which also included items capturing attitudes towards the importance of attractiveness. Notably, the single body gaze item used by Riemer et al. (2022) referred to a difficulty “not to gaze at women’s” body parts suggesting that sexually objectifying body gaze behavior may involve inhibitive difficulties.

Accordingly, and in a more deliberate effort to develop a standalone body biased gaze scale, Hollett et al. (2022) argued that body gaze behavior becomes maladaptive or pervasive, when the pattern of behavior is effortful, uninhibited, and contextually inappropriate. These aspects of sexually objectifying gaze were distilled into a five-item scale with evidence of good internal consistency and test–retest reliability. Construct validity was supported by positive correlations between pervasive body gaze scores and both automatic (eye tracking towards images of women) and explicit (victim blame) measures in heterosexual men. Importantly, the items used to capture pervasive body gaze do not refer to sexual or aggressive contexts thus avoiding admission to criminal acts or descriptions of intimate partner relations. While preliminary results suggest that pervasive body gaze is a potential marker for sexual objectification, further support for this measure would involve finding correspondence with implicit and affective markers of sexual objectification.

### Alternatives to Self-Reported Measurement of Sexual Objectification

Given the challenge of accurately capturing socially undesirable attitudes, self-reported instruments are sometimes complemented by other methods designed to detect aspects of sexual objectification. For instance, objectively measuring gaze behavior using eye tracking technology is an intuitive method for understanding how attentional resources are allocated when men visually appraise women. Indeed, several eye tracking studies have reported that men show attentional preferences towards women’s bodies but this is effect is most evident when the female imagery is sexually objectifying or reflects idealized body shapes (Gervais et al., 2013; Hollett et al., 2019, 2022; Nummenmaa et al., 2012). Importantly, eye tracking studies have also found that body biased gaze correlates positively with self-reported endorsement of sexual objectification attitudes and rape myths (Bareket et al., 2018; Hollett et al., 2022). While these findings are largely consistent with sexual objectification assumptions, the paradigms used to objectively capture gaze behavior often lack external validity. Specifically, gaze tasks often require contrived and highly controlled stimuli presentations to accurately capture automatic attentional patterns. These presentations can differ markedly from how women are encountered in the real world. Furthermore, eye tracking also cannot be easily deployed to large samples because high resolution infrared devices are typically required. Self-report instruments such as the pervasive body gaze scale offer some benefits over objective gaze measures because they can be deployed to large samples, and they provide an estimate of body gaze behavior from a dispositional perspective. Dispositional or trait measurement might be more generalisable across different contexts than measurements from the highly specific paradigms used in laboratory studies (Allport, 1937; Endler, 1988; Endler & Parker, 1992).

While direct measurement of visual attention has recently grown in popularity for making assumptions about potential socio-cognitive biases, implicit attention paradigms have been used for decades with similar objectives (Greenwald et al., 1998; Schimmack, 2021; Sriram & Greenwald, 2009). It is plausible to test for several sociocognitive biases derived from the sexual objectification framework using implicit tasks. For instance, that women are perceived as objects/nonhuman and exhibiting a tolerance of sexual assault. Indeed, several variants of implicit association tasks have been used to measure the propensity to exhibit sexually objectifying attitudes. For instance, an image-based Implicit Association Test (IAT) requiring participants to categorize objectified imagery of women and men using animal words or human words found that the weakest human associations were exhibited for the objectified female imagery (Vaes et al., 2011). Rudman and Mescher (2012) used two versions of a text-based implicit association test, one which captured automatic associations between women and objects, and one between women and animals. Faster categorisation of female concepts (e.g., she, her) with both objects (e.g., device, tool) and animals (e.g., hoof, snout) was significantly correlated with a self-reported rape proclivity score. Furthermore, multiple studies using an IAT requiring men to classify “rape” as good or bad found that higher associations between positive words and rape (e.g., rape-good) correlated with self-reported sexual coercion and subsequent sexual aggression (Hermann & Nunes, 2018; Nunes et al., 2013). These studies suggest that IATs may be useful for capturing biases that might accompany explicit sexually objectifying attitudes and behavior. Therefore, construct validity for explicit measures (e.g., self-report) could be partly achieved by yielding significant correlations with implicit association tasks which target sexually aggressive concepts.

Tolerance of sexual aggression towards women may also be measured via affective methods. Specifically, the general aggression model posits that aggressive cognition and affect may be accompanied by diminished empathy and desensitization to violence (Anderson & Bushman, 2018). Desensitization refers to a lower aversion to violence, often evidenced using attitudinal, affective or physiological measures (Read et al., 2016). That is, individuals who are desensitized to violence are expected to exhibit greater tolerance of violent stimuli (Funk et al., 2004). Given that sexual objectification research suggests that dehumanization, increased victim blame, and lower moral concern for women are potential mechanisms for explaining the mistreatment of women, it follows that affective measures may be useful for characterising a desensitization to victimized women (Bernard et al., 2015; Loughnan et al., 2010; Rudman & Mescher, 2012). Specifically, men who develop sexually objectifying attitudes towards women may also exhibit diminished affective responses towards stimuli of injured or victimized women. Indeed, evidence suggests that college men and incarcerated men who report sexually coercive behavior also exhibit empathic deficits, putting them at higher risk of perpetrating a sexual assault (DeGue & DiLillo, 2004; DeGue et al., 2010).

Image exposure tasks provide some support for a desensitization effect when making valence judgements of sexually victimized women. Specifically, Widman and Olson (2013) measured reaction time to appropriately label positive (e.g., awesome) or negative (e.g., gross) adjectives which followed exposure to an image of a male perpetrated sexual assault against a female. Participants who were faster to respond to positive adjectives than negative adjectives following exposure to these rape image primes also reported a higher frequency of perpetrated sexual assault. That is, men with a higher propensity to commit sexual assault were also quicker to assign positive valence when primed with imagery depicting a female victim of a sexual assault. This finding could be predicted by both sexual objectification and general aggression theories, but such image exposure paradigms are not readily administered, likely due to ethical concerns. More recently, Cogoni et al. (2018) found that participants exhibited lower self-reported positive emotional and neural responses in areas of the brain involved in empathy when rating the emotion of sexually objectified woman compared with a non-objectified woman. While these reaction time and neuroimaging studies have provided some insight into affective responses when processing sexually objectifying material, skin conductance (sweat activity) is another potentially useful method for understanding men’s affective responses towards women. Specifically, the skin conductance response (SCR) is a useful but non-specific measure of the initial stages of sympathetic nervous system activation, which may arise in response to novelty, personally relevant or salient stimuli (such as one's name), threat assessment, or the onset of emotional arousal (either positive or negative) (Boucsein, 2012). Importantly, SCR has been used previously to explore desensitization to violence by determining if exposure to violent stimuli can lead to dampened sympathetic nervous system activation during subsequent exposure to violence. For example, Carnagey et al. (2007) found that, compared to playing a non-violent video game, playing a violent video game lead to lower skin conductance in response to subsequently presented videos of real life violence. Similarly, Staude-Müller et al. (2008) demonstrated that following experimental exposure to a violent video game, men exhibited lower skin conductance in response to aversive still images. Both these studies demonstrate that SCR may be a useful affective marker for desensitization during image exposure paradigms. However, to our knowledge, no studies have used skin conductance as a potential marker for affective responses which might correspond with sexual objectification. Consistent with the female victim image exposure research by Widman and Olson (2013), we argue that dampened affective responses towards injured women may be a useful complement to implicit and self-report methods for understanding how men sexually objectify women.

### The Present Study

Reliable and valid measurement tools are critical to the advancement of any field and there is an emerging interest in developing new instruments to quantify sexual objectification with the capacity to predict sexual assault propensity (Gervais et al., 2018; Hollett et al., 2022; Riemer et al., 2022). To offer increased confidence in one of these emerging sexual objectification measures, we aimed to further validate a self-report measure of pervasive body gaze in heterosexual men by determining its association with implicit, physiological, and explicit (self-report) measures. To achieve this, we first measured SCR during exposure to female models in casual clothes and underwear with and without injuries to capture a physiological measure of desensitization. That is, we assumed that smaller increases in SCR during exposure to images of injured women in their underwear (relative to neutral imagery) would reflect a desensitization effect because such imagery would be considered less salient or emotional engaging to an individual who is at greater risk of sexually assaulting a woman. We also subsequently measured self-reported sexual arousal to these same images. A new version of a brief IAT was developed which is more conceptually related to sexual aggression than woman/animal IATs and less confronting than IATs using the term “rape”. Specifically, our IAT aimed to measure the strength of the association between the concepts of “erotic” and “rough” (relative to erotic and gentle). Ultimately, these methods were designed to balance ethical boundaries with practicality whilst extending on important contributions made by studies already exploring sexual aggression using image exposure and implicit based methodologies.

In line with assumptions that sexual assault is preceded and accompanied by a heightened tolerance or preference for rough sexual conduct, and diminished affective responses towards female victims we made the following predictions:

#### H1

It was expected that exposure to non-neutral images (underwear, injured, and underwear/injured) would elicit larger average skin conductance responses than the neutral images.

#### H2

It was expected that exposure to underwear images would elicit the highest self-reported sexual arousal compared to the neutral, injured, and underwear/injured images.

#### H3

Pervasive body gaze was expected to correlate positively with two rape myth victim blame subscales (She wanted it and She asked for it).

#### H4

Pervasive body gaze was expected to correlate positively with sexual assault perpetration (unwanted sexual advances).

#### H5

Pervasive body gaze was expected to correlate positively with implicit rough/erotic scores.

#### H6

Pervasive body gaze was expected to correlate *negatively* with physiological reactivity to the underwear and underwear/injured images, relative to neutral images. That is, those with high pervasive body gaze tendencies were expected to experience smaller increases in their physiological responses to images of injured women in underwear.

#### H7

Pervasive body gaze was expected to correlate positively with the subjective sexual arousal ratings of the underwear and underwear/injured images.

#### H8

Pervasive body gaze was expected to correlate positively with an estimate of violent pornography use.

## Method

### Participants

Self-reported heterosexual men (*N* = 110) were recruited from the university and surrounding communities. Participants ranged from 18 to 62 years (*M* = 28.31, SD = 9.94), and were mostly Caucasian (75%), followed by Asian (11%), African (3%), mixed (3%) or other (8%).

### Measures

#### Pervasive Body Gaze

Pervasive body gaze was assessed using the 5-item Pervasive Body Gaze Scale (Hollett et al., 2022), which was previously validated using eye tracking in heterosexual men. The scale items describe body gaze instances specifically towards women which are convert, uninhibited, effortful, and contextually inappropriate. Participants rated the items on a 5-point Likert scale from 1 (*strongly disagree*) to 5 (*strongly agree*), with the items averaged to yield a total score. Alpha for the pervasive body gaze score in the current sample was .84.

#### Victim Blame

Two subscales were taken from the IRMAS to assess victim blame attitudes (Payne et al., 1999). Specifically, the She Asked for It (SA; 8 items) and the She Wanted It (WI; 5 items) scales. Participants rated the items on a 7-point Likert scale from 1 (*not at all agree*) to 7 (*very much agree*), with the items of each subscale averaged to yield a separate score. Alphas for each subscale were .90 (SA) and .81 (WI).

#### Perpetration of Sexual Assault

The unwanted advances subscale from the Interpersonal Sexual Objectification Scale (Perpetration Version) was used to capture instances of sexual assault (Gervais et al., 2018). Because the four items refer to admission of non-consensual touching and harassment, it is essentially a measure of sexual assault perpetration, by definition in the US and Australia (Australian Bureau of Statistics, 2018; Office on Violence Against Women, 2020). Participants rated the items on a 5-point Likert scale from 1 (*Never*) to 5 (*Almost always*), with the items averaged to yield a total score. Alpha for the unwanted advances score in the current sample was .81.

#### Implicit Rough/Erotic Association

An adaptation of the Brief Implicit Association Test (BIAT; Sriram & Greenwald, 2009) measured the cognitive association between sex and aggression. Participants categorized erotic (e.g., sexy, arousing) or unerotic (e.g., gross, foul) words with either rough (e.g., smack, slap) or gentle (e.g., softly, careful) words across 4 blocks of 20 trials. The strength of the association between the erotic and rough words were computed using D, a specialized effect size measure unique to this task, with a range of − 2 to + 2 (see Greenwald et al., 2003; Sriram & Greenwald, 2009). Higher and positive D scores indicated a stronger association between the erotic and rough words and negative or lower D scores indicated a stronger association between the erotic and gentle words. The task was programmed and delivered using Inquisit Lab 5 (Inquisit, 2018).

#### Female Imagery

A professional photographer was contracted to take studio photos of six amateur female models who responded to an advertisement on a local talent platform. The models held a neutral facial expression for all the photos. A professional make-up artist applied the injuries. Four images were produced for each model, a neutral image (casual clothes), a sexualized image (underwear), and an injured version of both these images. The neutral image was used as a control (baseline) image to determine relative physiological reactivity to the other image types (injured, underwear, and injured/underwear). Due to differing bodily proportions across the models, image sizes were standardized using head dimensions (26 mm × 32 mm). Each image was approximately 58 mm × 222 mm (or 220 × 840 pixels) in total size.

##### Skin Conductance Response (SCR)

Tobii Pro Lab was used to present the female imagery and measure/filter skin conductance responses via a Shimmer3 GSR + unit, which records data at a sampling rate of 120 Hz. Event-related SCR data was measured in microsiemens (µS) and captured 1–5 s after each image onset, above a minimum amplitude threshold of 0.03 µS. Two reactivity scores were used for analysis. Firstly, and to perform a manipulation check, the average SCR reactivity (in microsiemens) across each of the four image sets were compared. This allowed us to determine whether the neutral imagery condition yielded the lowest SCR response relative to the other imagery conditions and confirm its suitability as a baseline comparison. Secondly, for the correlational analyses, difference scores were created by subtracting average SCR reactance to baseline (neutral) imagery from the average SCR reactance to the underwear, injured, and underwear/injured images. That is, three reactance *difference* scores were produced, one for underwear imagery, one for injured imagery, and one for underwear/injured imagery. Skin conductance responses have been described as a reliable index of affective responses in media research (Ravaja et al., 2006).

#### Subjective Image Arousal Ratings

Following the physiological and implicit tasks, participants were shown the same female imagery from the skin conductance procedure individually for a second time to make subjective ratings. Specifically, they were asked “How sexually arousing is this image?” with ratings of 1 (*very unarousing*) to 7 (*very arousing*). That is, higher ratings indicated the image had a higher capacity to sexually arouse. The arousal ratings for the images in each set were averaged to create separate scores for the neutral, underwear, injured, and underwear/injured images respectively.

#### Violent Pornography Use

Participants who reported they had used pornography in the last month (*N* = 107) were presented with a slider to estimate the percentage (0–100%) of this pornography that contained violence or suggested violence.

### Procedure

**Fig. 1:**
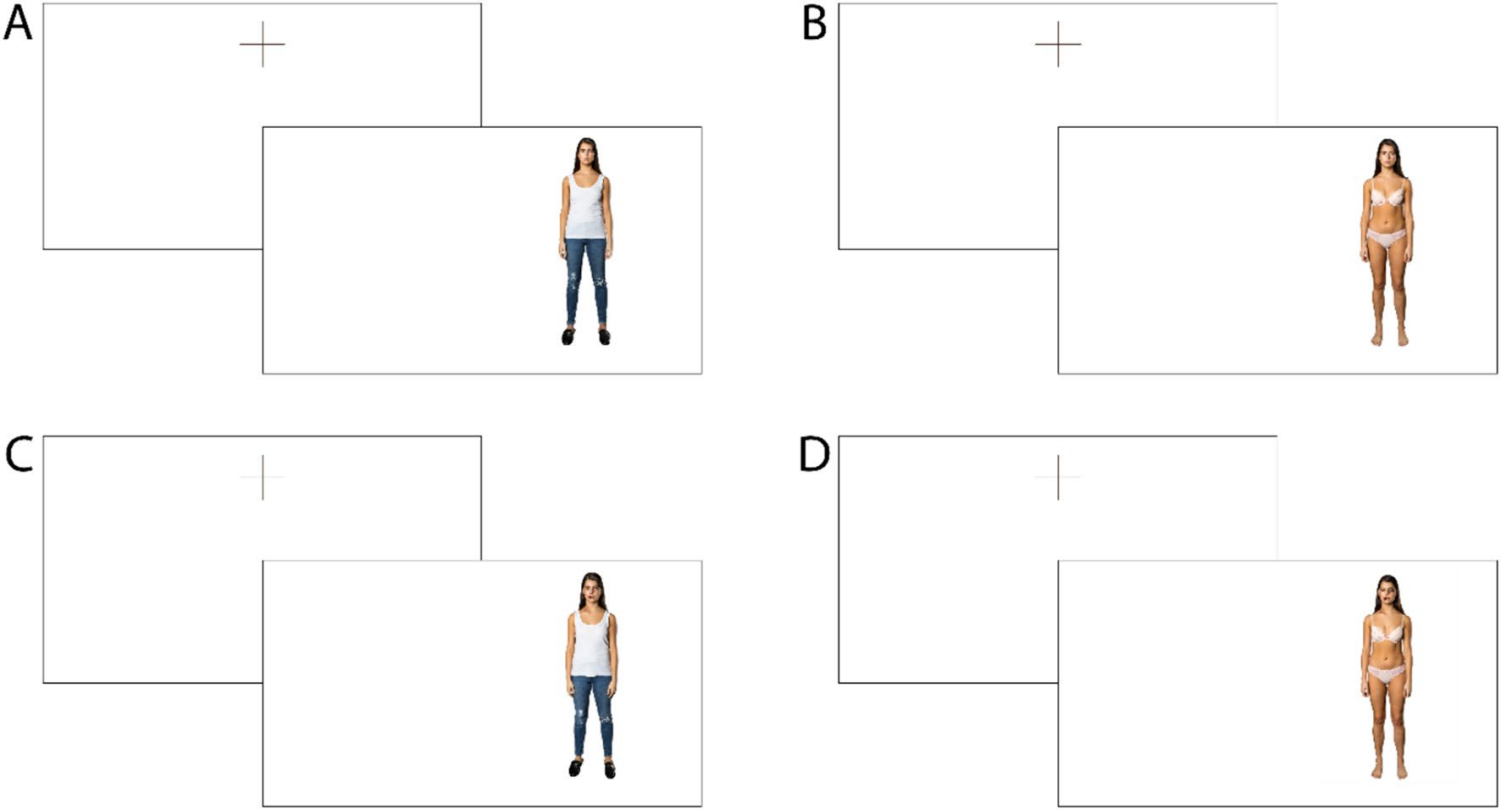
Examples of **A** Neutral, **B** Underwear, **C** Injured, and **D** Underwear/injured images

Participants were sought using university and community online and physical noticeboards for a study on “responses to sexual and aggressive material.” Participants were also recruited from an undergraduate research credit scheme. Respondents were sent study information prior to arriving at a university laboratory. Upon arrival and providing informed consent, participants had Shimmer GSR + electrodes attached using Velcro straps to the palmer surface of the index and middle fingers of their left hand. Due to the configuration of the equipment, available space and the simplicity of the image exposure task, we opted to attach the electrodes only to the left hand as this was least likely to be dominant and would prevent the need to be shifting the orientation of our equipment for each session. Participants were advised to keep their fingers slightly spread (to avoid contact between the straps) and as still as possible during the image exposure. Prior to image exposure, participants read on-screen instructions explaining that there would be a series of images shown and they should look at each image as they would normally look at a person. Participants were also informed that they would be asked to rate the images later in the session. The images were presented in four separate 42-s blocks. The first block contained the six control images (casually dressed and uninjured), the remaining blocks were presented in a counter balanced order and contained the six underwear (uninjured images), the six injured (dressed casually), and the six underwear/injured images. A minimum 10 s delay occurred between each image block to allow some opportunity for any elevated skin conductance reactivity to subside before the next presentation. Within each block, each image was presented in a random lateral location on the screen for 5 s each (totalling 30 s of each image type), preceded by a central fixation cross for 1 s and followed by a 1 s intertrial interval. See Fig. 1 for an illustration of the stimuli presentation (enlarged version available in the supplementary materials).

Following the skin conductance task, the electrodes were removed, and participants commenced the rough/erotic BIAT. While the BIAT was self-instructed, participants were first advised to read through the instructions carefully as the task can be tricky. Following the BIAT, participants completed a self-paced survey to collect the subjective image sexual arousal ratings and remaining self-report measures. Note that all data was collected anonymously behind a privacy curtain within our laboratory and the researcher left the room prior to each measurement. At the completion of the survey, participants were debriefed and remunerated (course credit or a $20 gift card). Each session lasted between 45 and 60 min.

#### Research Design and Analysis

**Fig. 2:**
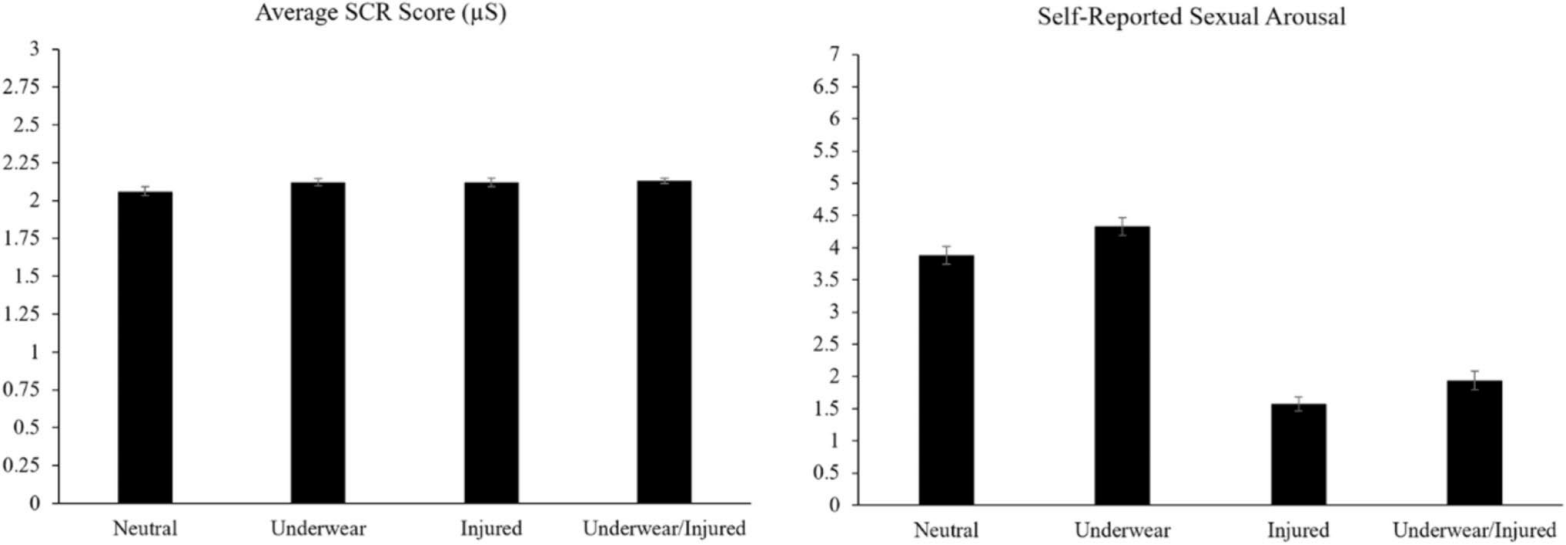
Means and 95% confidence intervals physiological subjective image reactivity measures

The current study used experimental within-subjects and correlational design elements. The within-subjects analyses involved comparing the physiological and subjective arousal ratings across the image types using two separate one-way repeated measures ANOVAs. Follow up paired-sample t-tests with an alpha adjustment for multiple comparisons (α = .01) were used to determine which image sets elicited the lowest and highest physiological and subjective arousal. Note that the 95% confidence intervals for the Fig. 2 within-subjects effects were calculated by removing the between-subjects variability (Cousineau, 2005).

The correlational analyses involved bivariate correlations between pervasive body gaze and each of the other variables. Correlations between multi-item self-report measures were then corrected to disattenuate for imperfect reliability (Crocker & Algina, 1986). The associations were interpreted according to effect size guidelines provided by Gignac and Szodorai (2016) for individual differences research. Specifically, *r* values of .10, .20, and .30 were considered relatively small, typical, and relatively large, respectively.

## Results

### Data Screening

Two participants opted not to complete the sexual assault questionnaire items (rape myth acceptance and unwanted advances items). Thirteen participants were excluded from skin conductance analyses due to data losses and unusually large (± 3 SD) reactivity responses. Note that no participants were excluded for failing a SCR non-response threshold because all participants elicited a minimum threshold response to every image in the exposure task. Finally, 22 participants were excluded from the BIAT analysis for low accuracy (< 70%). Due to differing sample sizes and the importance of retaining cases for maximum statistical power, varied samples were used for the self-report (*N* = 108–110), physiological (*N* = 97), and implicit (*N* = 88) correlational analyses. All variables were normally distributed (skew <|2|; kurtosis <|4|).

### Physiological and Subjective Reactivity to Female Imagery

To test that the image sets elicited the predicted differences in physiological arousal, the average SCR scores were compared for the neutral, underwear, injured, and underwear/injured images. Note that the sphericity assumption was violated so the Greenhouse–Geisser correction was applied. There were statistically significant differences amongst the SCR scores, *F*(2.49, 239.37) = 4.77, *p* = .005, *ηp*^2^ = .05. Specifically, the neutral images elicited a significantly lower SCR score when compared to the underwear images (*p* = .004, *d* = .30) and underwear/injured images (*p* = .001, *d* = .34), but not the injured images (*p* = .025, *d* = .23) when adjusting for multiple comparisons. See Fig. 2 for an illustration of the mean values and confidence intervals. Therefore, H1 was partially supported because the underwear and underwear/injured images elicited higher physiological arousal than the neutral images.

To test that the image sets elicited the predicted differences in subjective arousal, the average self-reported sexual arousal ratings were compared for the neutral, underwear, injured, and underwear/injured images. Note that the sphericity assumption was violated so the Greenhouse–Geisser correction was applied. There was a statistically significant difference amongst the subjective sexual arousal scores, *F*(1.94, 211.53) = 304.38, *p* < .001, *ηp*^2^ = .74. Specifically, the underwear images elicited a significantly higher sexual arousal score when compared to the neutral images (*p* < .001, *d* = − .48), underwear/injured images (*p* < .001, *d* = 1.72), and the injured images (*p* < .001, *d* = − 2.31). See Fig. 2 for an illustration of the mean values and confidence intervals. Therefore, the second hypothesis was supported because the underwear images elicited the highest sexual arousal compared to the other images sets.

### Correlational Analysis

The correlations between the self-report, implicit, and physiological measures with pervasive body gaze have been reported in Table 1. As seen in Table 1 there were significant and relatively large positive associations between pervasive body gaze and victim blame scales (she wanted it; she asked for it), which supported H3. While the positive association between pervasive body gaze and sexual assault perpetration was significant and supported H4, it was only a typical effect size. There was a significant and typical positive association between pervasive body gaze and the rough/erotic BIAT score, supporting H5. Of the physiological measures, both the underwear and the underwear/injured images reactivity scores were significantly negatively correlated with pervasive body gaze with typical and large effect sizes, respectively. Finally, pervasive body gaze was significantly and positively correlated with subjective sexual arousal ratings for the image sets containing injuries (underwear and casual clothes). While the correlation between pervasive body gaze and the injured image arousal ratings was only of a typical effect size, the correlation between pervasive body gaze and underwear/injured image arousal ratings yielded a relatively large effect size. Therefore, H7 was partially supported because one set of underwear images subjective arousal ratings was significantly positively correlated with pervasive body gaze. A full zero-order correlation matrix between all measures is available in the supplementary materials. Finally, H8 was supported as there was a typical positive correlation between pervasive gaze and violent pornography use.

**Table 1:** Pervasive gaze descriptive statistics and correlations

	Pervasive body gaze
Mean (SD)	2.71 (.94)
Bivariate correlations	
Sexual assault variables (*N* = 108)	
She wanted it	.38***
She asked for it	.49***
Sexual assault perpetration	.28*
Implicit variable (*N* = 88)	
Rough/erotic BIAT score	.28**
Physiological reactivity variables (*N* = 97)	
Injured images reactivity score	− .17
Underwear images reactivity score	− .21*
Underwear/injured images reactivity Score	− .34***
Self-reported arousal variables (*N* = 110)	
Neutral images	.09
Injured images	.24*
Underwear images	.18
Underwear/injured images	.36***
Violent pornography use estimate (*N* = 107)	.22*

*p < .05; **p < .01; ***p < .001; BIAT = Brief Implicit Association Test. Note that a full zero-order correlation matrix has been reported in the supplementary materials

## Discussion

To further support the potential utility of a self-reported measure of behavioral sexual objectification in heterosexual men, the current study examined associations between a brief measure of pervasive body gaze and other self-reported, implicit, and physiological markers of sexual assault propensity. Pervasive body gaze correlated significantly with self-reported victim blame and sexual assault perpetration measures, implicit associations between erotic and aggressive concepts and physiological responses to images of injured women. These results support our assertions that body gaze behavior is a potentially useful marker of deviant sexual objectification attitudes and affective states.

These results also show consistency with recent research showing that pervasive body gaze is correlated with rape myth acceptance attitudes in men (Hollett et al., 2022) but extends substantially on this prior work. Specifically, by showing that pervasive body gaze corresponds with implicit, affective, and explicit measures which relate to sexual assault, the present study provides convincing evidence of construct validity. That is, pervasive body gaze reflects a core manifestation of sexual objectification by men towards women. The results also align with prior work suggesting that sexual objectification and sexual coercion involves desensitization towards women via self-reported perceived suffering, moral concern, and empathy measures (DeGue & DiLillo, 2004; Loughnan et al., 2010, 2013). The present study adds physiological evidence to this framework and is the first study to our knowledge to explore sexual objectification processes using physiological measurements.

### Implications

Since objectification theory was formally described, gaze behavior has been widely recognised as an important feature of sexual objectification behavior (Fredrickson & Roberts, 1997; Gervais et al., 2013). However, empirical evidence supporting these theoretical assumptions has only recently begun to emerge with studies establishing that body gaze behaviors correspond with various sexually objectifying attitudes and behaviors (Gervais et al., 2018; Riemer et al., 2022). Eye tracking studies have been particularly valuable in establishing some of these assumptions (Bareket et al., 2018; Hollett et al., 2022). Importantly, the present study represents a convenient step forward for research on body gaze behavior without the need for eye tracking hardware. The brevity of the pervasive body gaze scale offers an attractive and efficient option for expanding our understanding of gaze behavior in both research and applied settings.

One potential application for the pervasive gaze scale may include estimating the extent to which body gaze inclinations change throughout the course of a prevention or intervention program. For instance, one objective during these programs is to enhance empathy towards women through greater focus on women’s social and emotional experiences during/following sexual assault (Foubert & Newberry, 2006; Gidycz et al., 2011; Stewart, 2014). If such strategies are successful, then men may exhibit a greater awareness of women’s social and emotional value (rather than sexual value) which might manifest as reduced inclinations to engage in body gaze behavior. That is, reductions in pervasive body gaze might be one useful marker of intervention efficacy. Indeed, if interventions reduce cognitive and attentional biases towards women’s bodies, then underlying attitudes and impulses which facilitate sexual assault may also be reduced. Pervasive body gaze measurement may also be used as an early risk marker for sexual assault propensity. Early intervention and education in high-risk settings, such as colleges, are considered critical in the prevention of sexual assault (Gidycz et al., 2011; Streng & Kamimura, 2015). The brevity of the pervasive body gaze scale lends itself well to inclusion within large scale screenings in settings such as colleges.

### Limitations and Strengths

While we propose some practical uses for the pervasive body gaze scale optimistically, we acknowledge that such uses are limited to the cultural context in which the scale has been validated. That is, cross cultural validation is needed to confirm that the psychometric properties are acceptable across a broader representation of people. Certainly, cultural differences in eye contact norms may play a substantial role when interpreting gaze behavior. As such, variations to, or new, scale items might be required to detect nuances in sexually objectifying gaze across cultures. For example, evidence suggests that higher degrees of eye contact is expected by those adhering to Western cultures, whereas some Eastern cultures, such as Japan, expect and prefer lower degrees of eye contact (Akechi et al., 2013; Uono & Hietanen, 2015). Therefore, higher levels of body gaze in some cultures might be motivated by an attempt to avoid eye contact rather than reflecting sexually objectifying attitudes. We also note that sexual orientation and target gender may further complicate the interpretation of gaze behavior.

Several other limitations also impede the conclusions of the current investigation. For instance, we only included a narrow range female imagery in the image exposure task, mostly reflecting young Caucasian women. Note that the imagery for this study was produced at a relatively high cost, which prevented the development of a broader range of images (e.g., age and ethnicity). We recognise, however, the importance of using a more diverse (and representative) range of female subjects when examining physiological responses. With emerging artificial intelligence image generation capabilities, we expect that low cost and high-quality stimuli of human subjects will be readily available for future research on sexual objectification (Boyd et al., 2023; Pataranutaporn et al., 2021). Importantly, we lacked reliable estimates of prior exposure to sexually objectifying media content which are valuable for understanding individual differences in the development of both physiological and implicit tendencies. For instance, while our single-item violent pornography use estimate correlated modestly with pervasive gaze (*r* = .22) and the rough/erotic IAT (*r* = .25), it did not correlate with any SCR measures, suggesting a more comprehensive instrument would be useful in future. Finally, we recognise that research exploring contentious topics and socially undesirable behaviors invites a higher risk of response bias on self-report instruments. While we attempted to maximise the anonymity and privacy of the data collection to diminish these issues, we concede that our conclusions should be interpreted with the awareness that elevated measurement error may be present within the data.

Despite the limitations, the present study represents an important step forward in the validation of a self-report measure of sexual objectification. Our use of multiple measurement techniques to estimate sexual assault propensity separates our work from similar recent studies attempting to validate self-report instruments for sexual objectification which have relied primarily on single measurement techniques and online deployment (Gervais et al., 2018; Riemer et al., 2022). It is especially important to explore controversial topics using complementary implicit and psychophysiological techniques rather than self-report alone due to potential response biases. We also note that the pervasive body gaze scale was previously subjected to test–retest reliability and concurrent eye tracking validation, offering a rigorous psychometric evaluation. As such, the current work replicates and meaningfully extends the conclusions of Hollett et al. (2022) regarding pervasive body gaze behavior.

### Recommended Research and Conclusions

A desirable next step in this research agenda would be to implement the pervasive body gaze scale into an applied setting such as prevention or intervention programs with college students and sex offenders for instance. Collection of body gaze data within such programs would contribute to further validating the instrument whilst offering the potential for longitudinal analysis with sexual assault-related outcomes. Another important objective for future research would be to develop gaze intervention tasks which deliberately disrupt body gaze towards women and encourage face gaze behavior. Computerized attentional bias modification paradigms such as those developed by researchers for reducing threat bias in anxious individuals and avoiding alcoholic stimuli in alcoholics could be leveraged for this purpose (e.g., MacLeod & Mathews, 2012; Rinck et al., 2018).

Taken together with the findings of Hollett et al. (2022), we have now provided a comprehensive construct validation of the pervasive body gaze scale. Importantly, our methods and analyses have produced theoretically predictable results, in accordance with theories of aggression and sexual objectification (Anderson & Bushman, 2002, 2018; Fredrickson & Roberts, 1997; Szymanski et al., 2010). We hope that future researchers and practitioners will benefit from using our methodologies to continue exploring the mechanisms which contribute to the sexual objectification of women.

## Supplementary Information

Below is the link to the electronic supplementary material.

Supplementary file1 (DOCX 775 kb)

## Data Availability

The ethics approval for this research does not allow data sharing. However, the first author can be contacted if there are any queries directly relating to the data.
