# Supplementary material for: Evidence That Pervasive Body Gaze Behavior in Heterosexual Men Is a Social Marker for Implicit, Physiological, and Explicit Sexual Assault Propensities
Source: Arch Sex Behav. 2024 Jul 24;53(10):3795–807. doi: 10.1007/s10508-024-02953-y (PMC11588823; doi:10.1007/s10508-024-02953-y)
Supplement: Supplementary file 1 — Supplementary file1 (DOCX 775 kb) [file 10508_2024_2953_MOESM1_ESM.docx]

**Table 2.**

*Zero-order Pearson Correlations between All Variables*

| Variable | 1 | 2 | 3 | 4 | 5 | 6 | 7 | 8 | 9 | 10 | 11 | 12 | 13 | 14 | 15 | | 16 |
| --- | --- | --- | --- | --- | --- | --- | --- | --- | --- | --- | --- | --- | --- | --- | --- | --- | --- |
| 1. Pervasive body gaze | - |  |  |  |  |  |  |  |  |  |  |  |  |  | |  |  |
| 2. RMA - She wanted it | **.31** | - |  |  |  |  |  |  |  |  |  |  |  |  | |  |  |
| 3. RMA - She asked for it | **.42** | **.68** | - |  |  |  |  |  |  |  |  |  |  |  | |  |  |
| 4. Unwanted sexual advances | **.23** | .13 | **.30** | - |  |  |  |  |  |  |  |  |  |  | |  |  |
| 5. Rough/Erotic BIAT score | **.28** | .16 | .13 | -.11 | - |  |  |  |  |  |  |  |  |  | |  |  |
| 6. Average SCR – Neutral | **-.29** | -.06 | -.13 | -.09 | .12 | - |  |  |  |  |  |  |  |  | |  |  |
| 7. Average SCR – Underwear | **-.30** | -.05 | -.13 | -.09 | .10 | **.99** | - |  |  |  |  |  |  |  | |  |  |
| 8. Average SCR - Injured | **-.31** | -.06 | -.14 | -.09 | .10 | **.99** | **.99** | - |  |  |  |  |  |  | |  |  |
| 9. Average SCR – Underwear/Inj. | **-.32** | -.07 | -.14 | -.09 | .09 | **.99** | **.99** | **1.00** | - |  |  |  |  |  | |  |  |
| 10. SCR Reactivity - Underwear | **-.21** | .04 | -.05 | -.01 | -.14 | **.26** | **.37** | **.33** | **.33** | - |  |  |  |  | |  |  |
| 11. SCR Reactivity - Injured | -.17 | .00 | -.06 | -.03 | .07 | .13 | .19 | **.28** | **.22** | **.55** | - |  |  |  | |  |  |
| 12. SCR Reactivity – Underwear/Inj. | **-.34** | -.11 | -.13 | -.03 | -.15 | **.24** | **.31** | **.35** | **.36** | **.66** | **.76** | - |  |  | |  |  |
| 13. Sexual arousal - Neutral | .09 | -.11 | -.05 | .07 | **-.21** | -.10 | -.10 | -.10 | -.10 | -.01 | .00 | -.02 | - |  | |  |  |
| 14. Sexual arousal - Underwear | .18 | -.06 | .03 | .14 | -.03 | -.04 | -.05 | -.04 | -.05 | -.13 | -.04 | -.07 | **.64** | - | |  |  |
| 15. Sexual arousal - Injured | **.24** | .19 | **.20** | .01 | .11 | .07 | .06 | .05 | .04 | -.06 | -.14 | **-.23** | **.26** | .11 | | - |  |
| 16. Sexual arousal – Underwear/Inj. | **.36** | **.26** | **.32** | .09 | .11 | .01 | .00 | -.01 | -.01 | -.07 | -.11 | -.18 | **.29** | .18 | | **.81** | **-** |
| 17. Violent Pornography Use | **.22** | **.36** | .10 | .07 | **.25** | .10 | .08 | .09 | .08 | -.13 | -.03 | -.13 | -.12 | .00 | | .16 | .12 |

*Note.* Significant (*p* < .05) effect sizes in boldface; RMA = Rape myth acceptance; SCR = Skin conductance response; BIAT = Brief implicit association task.

**Figure 3.**

*Enlarged Example Illustrating Each Image Exposure Condition, Neutral, Underwear, Injured and Underwear/Injured Imagery Respectively.
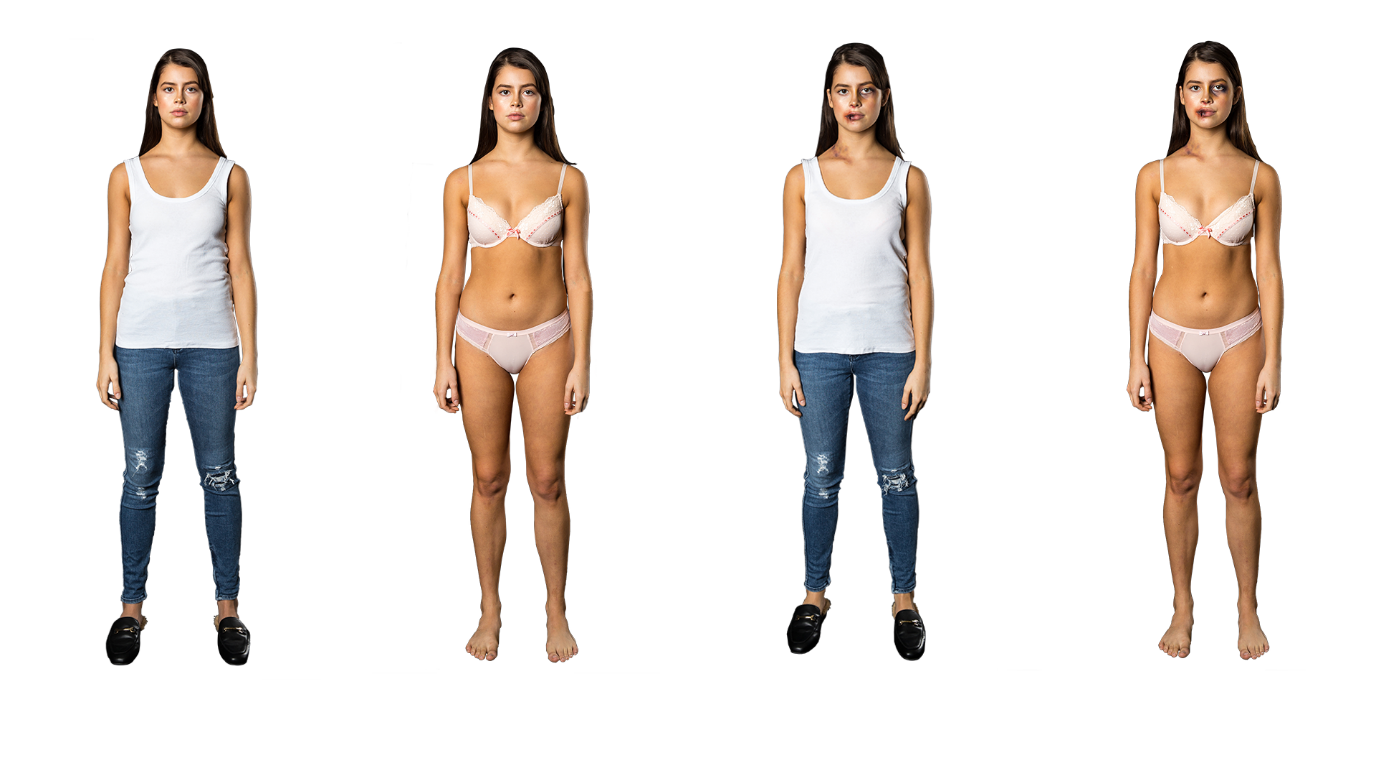
*
